# Autophagy is activated in systemic lupus erythematosus and required for plasmablast development

**DOI:** 10.1136/annrheumdis-2013-204343

**Published:** 2014-01-13

**Authors:** Alexander J Clarke, Ursula Ellinghaus, Andrea Cortini, Amanda Stranks, Anna Katharina Simon, Marina Botto, Timothy J Vyse

**Affiliations:** 1 Medical and Molecular Genetics and Division of Immunology, Infection, and Inflammatory Disease, King's College London, London, UK; 2 Nuffield Department of Clinical Medicine and Translational Immunology Laboratory, NIHR BRC, University of Oxford, Oxford, UK; 3 Department of Medicine, Centre for Complement and Inflammation Research, Imperial College London, London, UK

**Keywords:** B cells, Systemic Lupus Erythematosus, Autoimmunity

## Abstract

**Background:**

Autophagy has emerged as a critical homeostatic mechanism in T lymphocytes, influencing proliferation and differentiation. Autophagy in B cells has been less studied, but genetic deficiency causes impairment of early and late developmental stages

**Objectives:**

To explore the role of autophagy in the pathogenesis of human and murine lupus, a disease in which B cells are critical effectors of pathology.

**Methods:**

Autophagy was assessed using multiple techniques in NZB/W and control mice, and in patients with systemic lupus erythematosus (SLE) compared to healthy controls. We evaluated the phenotype of the B cell compartment in Vav-Atg7^−/−^ mice in vivo, and examined human and murine plasmablast formation following inhibition of autophagy.

**Results:**

We found activation of autophagy in early developmental and transitional stages of B cell development in a lupus mouse model even before disease onset, and which progressively increased with age. In human disease, again autophagy was activated compared with healthy controls, principally in naïve B cells. B cells isolated from Vav-Atg7^F/F^ mice failed to effectively differentiate into plasma cells following stimulation in vitro. Similarly, human B cells stimulated in the presence of autophagy inhibition did not differentiate into plasmablasts.

**Conclusions:**

Our data suggest activation of autophagy is a mechanism for survival of autoreactive B cells, and also demonstrate that it is required for plasmablast differentiation, processes that induce significant cellular stress. The implication of autophagy in two major pathogenic pathways in SLE suggests the potential to use inhibition of autophagy as a novel treatment target in this frequently severe autoimmune disease.

## Introduction

Autophagy is a highly conserved mechanism for the survival of cells during times of metabolic stress. Autophagosomes form at points of contact between the endoplasmic reticulum (ER) and mitochondria, and two ubiquitin-like conjugation systems (Atg12 and Atg8/LC3) extend a double-membraned autophagosome to sequester a portion of cytoplasm, which then fuses with the lysosome degradation pathway.^1^ ^2^

Its role as a key factor in lymphocyte biology has emerged only over the last few years.^3^ ^4^ Autophagy is required for the normal development of B and T lymphocyte populations,^5–8^ provides metabolic support to proliferating lymphocytes,^9^ ^10^ and is activated on B cell receptor (BCR) signalling in the absence of co-stimulation.^11^ ^12^

Systemic lupus erythematosus (SLE) is a potentially fatal autoimmune disease characterised by failure of multiple tolerance checkpoints, leading to the escape and proliferation of autoreactive B cells,^11^ ^13^ through mechanisms which are poorly understood.

Indirect evidence for the role of autophagy in SLE comes from genetic association studies implicating variants in the region of the autophagy gene *ATG5*,^14^ ^15^ which affect its expression in B lymphocytes,^16^ ^17^ and a therapeutic benefit in MRL/lpr mice from the peptide P140, which modulates autophagy.^3^ ^4^

To date, autophagy in SLE T cells has been examined and found to be activated in the naïve CD4^+^ T cell compartment by Alessandri *et al*,^5–8^ and the total CD4^+^ population by Gros *et al*.^9^ ^10^ However, autophagy in B cells, which are of fundamental importance in SLE, the hallmark of which is the production of pathogenic high affinity autoantibodies,^11^ ^12^ has not yet been studied in human autoimmune disease.

In this study, we demonstrate enhanced autophagy in murine and human lupus B cells, and that autophagy is required for B cell survival and maturation. Intervention in autophagy provides a potentially new therapeutic avenue for SLE.

## Materials and methods

### Mice

Female NZB/W_F1_ (NZB/W) and C57BL/6 (B6) mice were obtained from Charles River, UK. Atg7^Flox/Flox^ mice (a kind gift from Maasaki Komatsu, Tokyo Metropolitan Institute of Medical Science, Japan) were crossed with Vav-iCre (a kind gift from D Kioussis, London, UK) to obtain Vav-iCre; Atg7^Flox/Flox^.^11^ ^13^

### Patients

A total of 43 consecutive patients meeting the revised American College of Rheumatology (ACR) criteria for the definition of SLE^14^ ^15^ were recruited from hospitals of King's Health Partners following informed consent and with ethical approval. The mean age of the patients was 36, with a male:female (M:F) ratio of 0.12. Patients were excluded if they had received rituximab therapy within the last year, or intravenous methylprednisolone within the preceding month. SELENA-SLEDAI scores were calculated.^18^ Healthy controls were recruited from King's College London. The mean age of the controls was 33 years, with an M:F ratio of 0.18. We found no statistical association between age or sex and autophagy measures in the control group. Patient characteristics are listed in online supplementary table S1.

### Cell isolation and culture

Human peripheral blood mononuclear cells were separated from whole blood using Ficoll density centrifugation. CD19^+^ B cells were isolated using negative selection (DynaBeads Untouched B cell Isolation Kit, Invitrogen, UK), with routine purity >95%. Cell culture was performed in RPMI 1640 (Invitrogen, UK) supplemented with 10% fetal calf serum (FCS), 2 mM L-glutamine and 100 U/mL penicillin/streptomycin (all from Invitrogen, UK). For human plasmablast differentiation, B cells were stimulated with ODN2006 (5 μM) (Invivogen, USA), IL-10 (50 ng/mL) and IL-15 (10 ng/mL) (PeproTech, USA), and monoclonal mouse anti-CD40 L (1 μg/mL) (clone 82111, R&D Systems). Alternatively, human B cells were stimulated with goat anti-IgM F(ab)_2_ fragment (5 μg/mL) (Stratech, USA), monoclonal mouse anti-CD40 L (1 μg/mL), or recombinant human interferon-α2a (5000 U/mL) (Peprotech). Murine B cells were isolated by negative selection (Miltenyi, Germany), with a routine purity of >95%, and stimulated with lipopolysaccharide 10 μg/mL (0111:B4) (Sigma, UK) and IL-4 (10 ng/mL). Autophagy was inhibited by 3-methyladenine (5 mM), bafilomycin A_1_ (100 nM) or chloroquine (10 μM) (all Sigma, UK).

### Flow cytometry

In longitudinal experiments, cytometer settings were standardised using BD Cytometer Setup and Tracking Beads (BD Biosciences). Flow cytometry was performed on BD Canto II or Fortessa instruments. Cell viability was assessed with Live/Dead Green (Invitrogen, UK), or Fixable Viability Dye (eBioscience, UK).

Intracellular staining for p62 was performed following fixation and permeabilisation with BD Fix/Perm kit I (BD Biosciences). In human experiments, mouse monoclonal anti-p62-Alexa Fluor 647 (1 μg/mL) (clone 5F2, MBL, Japan) was used. Mouse cells were incubated with monoclonal rabbit anti-p62 1:500 (clone D10E10) (Cell Signaling Technology, USA), washed, and then incubated with DyLight 649-goat anti-rabbit secondary antibody (Abcam, UK).

For mitochondrial staining, cells were incubated in full media at 37°C for 30 min in the presence of 100 nM Mitotracker Deep Red FM and 100 nM Mitotracker Green (both Invitrogen, UK). Cells were washed and then surface stained.

Cell proliferation was measured by staining cells at room temperature for 10 min with 1 μM carboxyfluorescein diacetate succinimidyl ester (CFSE) (eBioscience, UK). Annexin V staining was performed using Annexin V-PE (eBioscience, UK).

### Multispectral imaging flow cytometry

For each sample, 5×10^6^ cells were used. Cells were stained for viability with Fixable Viability Dye (eBioscience, UK), then Fc receptors were blocked (mouse: anti-CD16/CD32 (clone 9); human: Fc Receptor Binding Inhibitor, both from eBioscience, UK). Cells were surface stained using antibodies listed above, then fixed and permeabilised with the BD Fix/Perm kit I (BD Biosciences). For mouse cells, samples were blocked with 10% goat serum, incubated with rabbit polyclonal anti-LC3 1:400 (Novus Bio, USA), washed, and incubated with Alexa Fluor 488-goat anti-rabbit secondary antibody (Invitrogen, UK). For human cells, samples were incubated with mouse monoclonal anti-LC3-FITC 1:400 (clone 2E6) (Enzo Life Sciences) and mouse monoclonal anti-active capase-3-PE (C92-605, BD Bioscience). Multispectral imaging flow cytometry (MIFC) was performed on an Amnis ImageStream^X^ instrument. Up to 2.5×10^5^ images were acquired per sample. Cells were gated on aspect ratio to include only singlets, and the gradient root-mean-square feature to include focused cells. Non-viable and apoptotic cells were excluded from analysis based on signal intensity. Using a spot count mask based on the FITC channel of the instrument, the number of LC3^+^ punctae per cell were quantified.

### Autophagosomotropic dye staining

Up to 1×10^6^ cells were incubated in RPMI (supplemented with 10% FCS and 2 mM L-glutamine but not antibiotics) at 37°C for 30 min in the presence of 0.25 μL/mL Cyto-ID Green Autophagy Detection Reagent (Enzo, UK). Cells were subsequently analysed by flow cytometry. Data are expressed as the mean fluorescence intensity of CytoID divided by the mean forward scatter of the cells, to correct for difference in cell sizes.

### ELISA

Mouse immunoglobulins were detected using an immunoglobulin isotyping kit (eBioscience, UK), according to standard techniques.

### Statistics

Statistics were calculated using either GraphPad Prism 5, or the R package. Student's t test, or analysis of variance with Tukey's post-test was used as appropriate. Regression modelling of medication use and autophagosome count was performed with R. Results were considered significant if p<0.05.

## Results

### Autophagy is increased in the B lymphocytes of the NZB/W F_1_ murine lupus model

**Figure 1:**
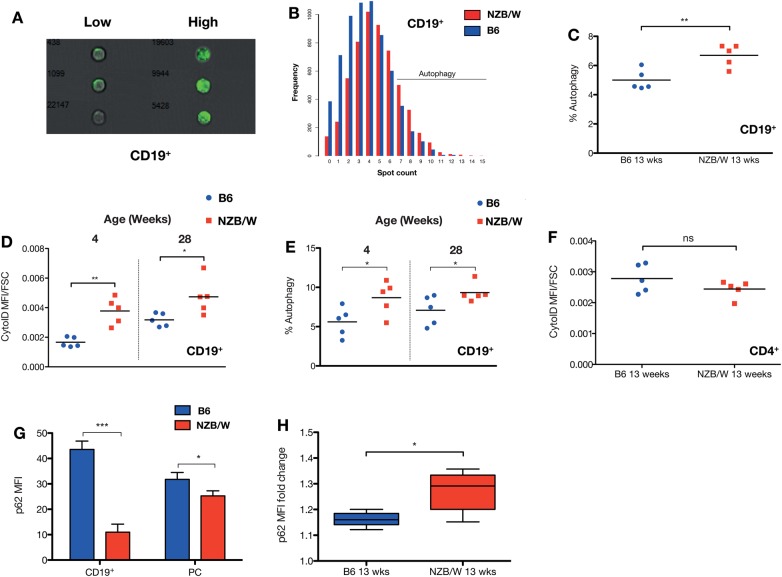
Autophagy in murine lupus. Autophagy in splenic NZB/W CD19^+^ B cells was compared with control C57BL/6 mice. (A) Representative multispectral imaging flow cytometry (MIFC) images of B cells with high and low numbers of LC3^+^ autophagosomes. (B) Example distributions of LC3^+^ punctae in diseased versus control mice at 13 weeks of age, with gating of autophagy-positive cells (defined as ≥7 spots per cell). There are increased numbers of LC3^+^ autophagosomes in the CD19^+^ B cells of NZB/W compared with control mice at 13 weeks (post-disease onset) (C). The number of autophagosomes measured using autophagosomotropic dye (D) or MIFC (E) increases with age, but a difference between the mice is present even at 4 weeks, before disease has developed. There was no difference in autophagosomotropic dye uptake in CD4^+^ T cells, at 13 weeks (F). Each data point represents an individual mouse. The horizontal bars denote mean. (G) There is decreased basal p62 expression, measured by intracellular FACS in splenic CD19^+^ B and CD19^+/−^CD138^+^ plasma cells in 13-week-old NZB/W mice. To demonstrate accumulation of the autophagic substrate p62, isolated splenocytes were incubated in complete RPMI with 100 nM bafilomycin A_1_ for 3 h (H). n=5 mice per group. Fold change in p62 MFI compared with untreated cells is shown. *p<0.05; p<0.01; ***p<0.001.

The New Zealand (black×white) F_1_ hybrid mouse (NZB/W) develops spontaneous autoimmune disease by 12 weeks of age, which shares many characteristics with human SLE, such as the production of high-affinity anti-dsDNA IgG antibodies, glomerulonephritis and female sex bias.^19–21^ We used MIFC to quantify the number of LC3^+^ autophagosomes in CD19^+^ B cells in 13-week-old female NZB/W mice compared with C57BL/6 (B6) controls of similar age and sex. In this technique, following immunofluorescent intracellular staining of endogenous LC3, high-resolution images of >100 000 individual cells in flow were captured and the number of LC3^+^ positive punctae (spot count) were calculated, following background subtraction. Figure 1A shows representative images of B cells with high and low numbers of LC3^+^ spots, and 1B typical distributions of spot count between the mice. There were significantly more autophagosomes (LC3^+^ punctae) in splenic CD19^+^ B cells in NZB/W mice compared with controls (figure 1C).

To quantify autophagosomes by an alternative technique, we used the novel amphiphilic autophagosome tracer dye CytoID, which co-localises with LC3 and has negligible non-specific staining of lysosomes.^22^ There was an increase in CytoID uptake in splenic total CD19^+^ B cell populations, but no difference in splenic CD4^+^ T cells (figure 1D,E). Autoimmunity in the NZB/W mouse model is age dependent, with autoantibodies developing after 8 weeks of age.^19^ To determine whether autophagy was differentially elevated in young, pre-disease mice, we examined NZB/W mice at 4 and 28 weeks of age. We found that even at 4 weeks, before the onset of disease, there was increased autophagy in the NZB/W mice compared with the control group (figure 1D,E). This difference was maintained at a similar level at 28 weeks.

Autophagy is a dynamic process and the possibility exists that an accumulation of autophagosomes may be due to a defect in clearance by lysosomal fusion.^23^ To evaluate autophagic flux, we examined the level and dynamics of the autophagosome adapter protein p62. p62 is degraded principally by autophagy, and its level is inversely correlated with autophagic flux.^24^ The level of basal p62 expression was significantly lower in the CD19^+^ B cell and plasma cell populations of the NZB/W mice compared with the control group (figure 1G,H). To measure autophagic flux, bafilomycin A_1_ was used to block lysosomal degradation of p62. Accumulation of p62 is expected if there is normal autophagosome–lysosome fusion. There was a greater accumulation of p62 in the NZB/W splenic B cells following this treatment, indicating higher autophagic flux.

**Figure 2:**
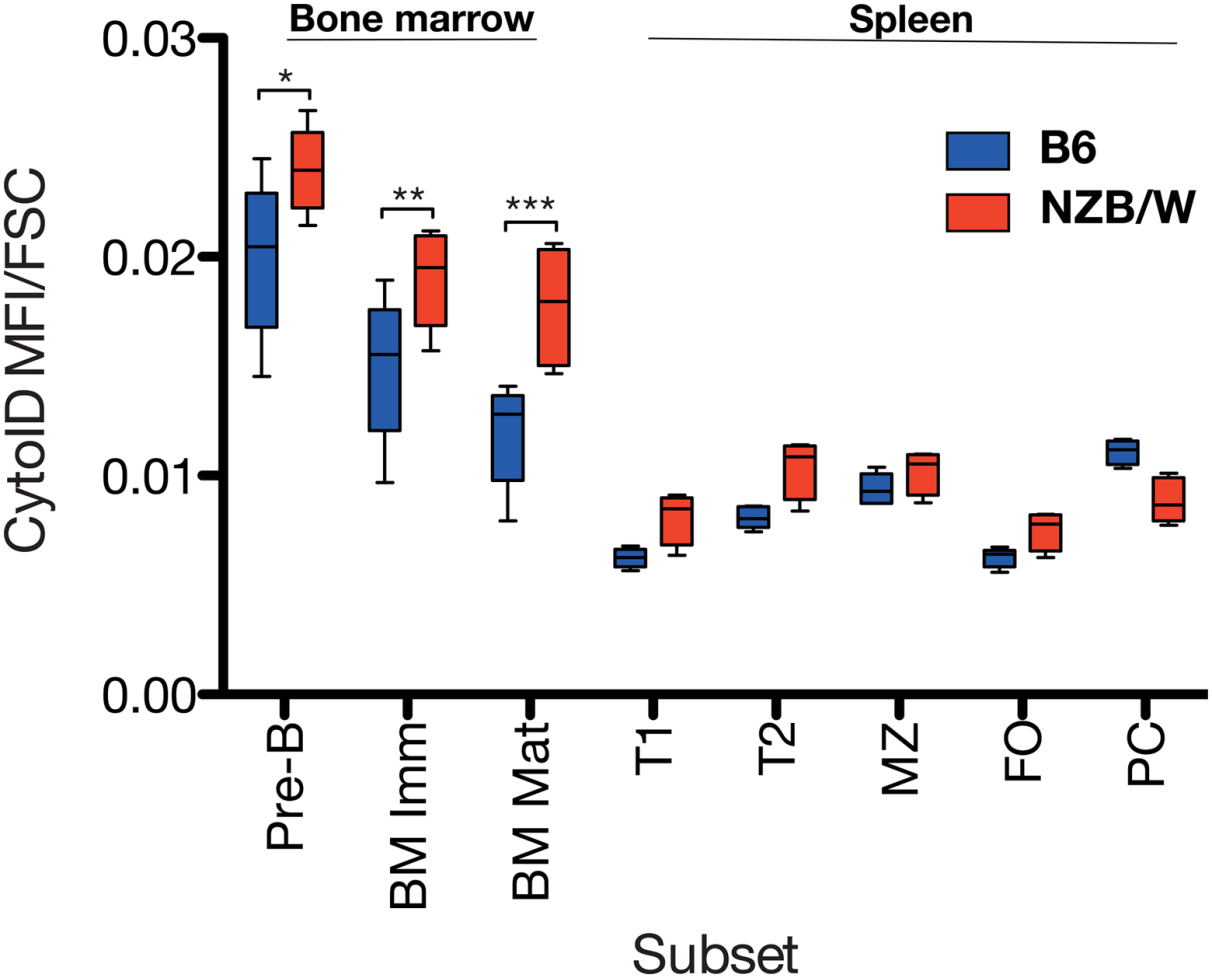
Autophagosome density in mouse B cell subsets. Distribution of CytoID MFI in B cell subsets from bone marrow (BM) and spleens of 13-week-old NZB/W mice and age matched controls. Pre-B (CD19^+^IgM^−^IgD^−^), immature B (BM Imm, CD19^+^IgD^−^IgM^+^), mature B (BM Mat, CD19^+^IgD^+^IgM^+^), T1 (CD19^+^IgM^+^ IgD^−^CD23^−^CD21^−^), T2 (CD19^+^IgM^+^ IgD^+^CD23^+^CD21^+^), marginal zone (MZ, CD19^+^IgM^+^IgD^−^CD23^−^CD21^hi^), follicular (FO, CD19^+^IgM^−^IgD^+^CD23^hi^CD21^+^), and BM plasma cell (CD19^+/−^CD138^+^) subsets are illustrated. n=5 mice per group. Box and whisker plots denote maximum and minimum, IQR, and median. *p<0.05; **p<0.01; ***p<0.001.

More detailed B cell immunophenotyping of the bone marrow (BM) and spleen (figure 2) revealed that in both groups of mice, autophagy was markedly increased in the BM compared with the periphery, and there was a reduction in autophagy in progressive early developmental stages, from pre-B, to immature, and then to mature BM B cells. Autophagy was generally elevated in the B cells of NZB/W mice, but most markedly so in the BM mature B cell population.

Autoimmunity in the NZB/W mouse model is age dependent, with autoantibodies developing after 8 weeks of age.^19^ To determine whether autophagy was differentially elevated in young, pre-disease mice, we examined NZB/W mice at 4 and 28 weeks of age. We found that even at 4 weeks, before the onset of disease, there was increased autophagy in the NZB/W mice compared with the control group (figure 1D,E). This difference was maintained at a similar level at 28 weeks. These data therefore implicate autophagy in the constellation of genetically determined abnormalities present in the NZB/W mouse strain.

### Autophagy is increased in B and T cells in human SLE, and is correlated with disease activity

**Figure 3:**
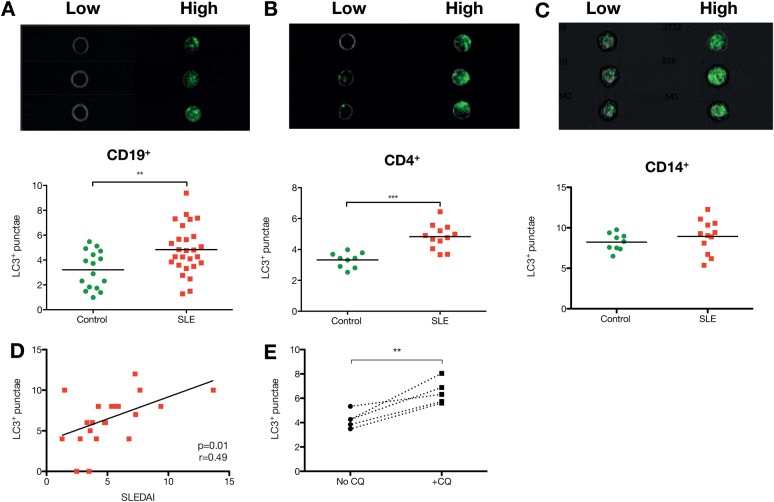
Autophagy in systemic lupus erythematosus (SLE) patients versus healthy controls. (A–C) Representative images of CD19^+^ B cells, CD4^+^ T cells, and CD14^+^ monocytes with high and low numbers of LC3+ punctae, and comparison with healthy controls. CD19^+^ B cell LC3^+^ puncta count is correlated with SELENA-SLEDAI disease activity index (D). (E) Increased autophagic flux in SLE patients. Isolated CD19^+^ B cells from patients were incubated with 10 μM chloroquine in complete RPMI media for 2 h, and then analysed by multispectral imaging flow cytometry. Each point represents one individual patient or healthy donor. **p<0.01; ***p<0.001.

Using MIFC, more LC3^+^ punctae were found in both CD19^+^ B and CD4^+^ T cells in SLE, but not monocytes, compared with healthy control subjects (figure 3A–C). The B cell LC3-BDI^+^ punctal count was positively correlated with the SELENA-SLEDAI disease activity score (figure 3D). A potentially confounding factor could be the use of hydroxychloroquine, which might increase autophagosome count by blocking fusion with lysosomes, however there was no significant correlation between LC3-BDI and use of this drug (p=0.52). Similarly, we found no association with specific immunosuppressant medication use, although there was a trend towards correlation with prednisolone dose (r=0.39, p=0.08). To more directly demonstrate intact autophagic flux in SLE, isolated CD19^+^ B cells were incubated with chloroquine, an alternative lysosomal acidification inhibitor, and LC3 punctae measured by MIFC. There was a significant increase in autophagosome number following this, indicating active autophagic flux (figure 3E). Finally, to confirm intact autophagosome–lysosome fusion, we examined co-localisation between lysosomes and autophagosomes in CD19^+^ B cells using MIFC (see online supplementary figure S1).^25^ There was no fusion defect in SLE patients compared with controls, but a non-statistically significant trend towards increased co-localisation, supporting the inhibitor data.

**Figure 4:**
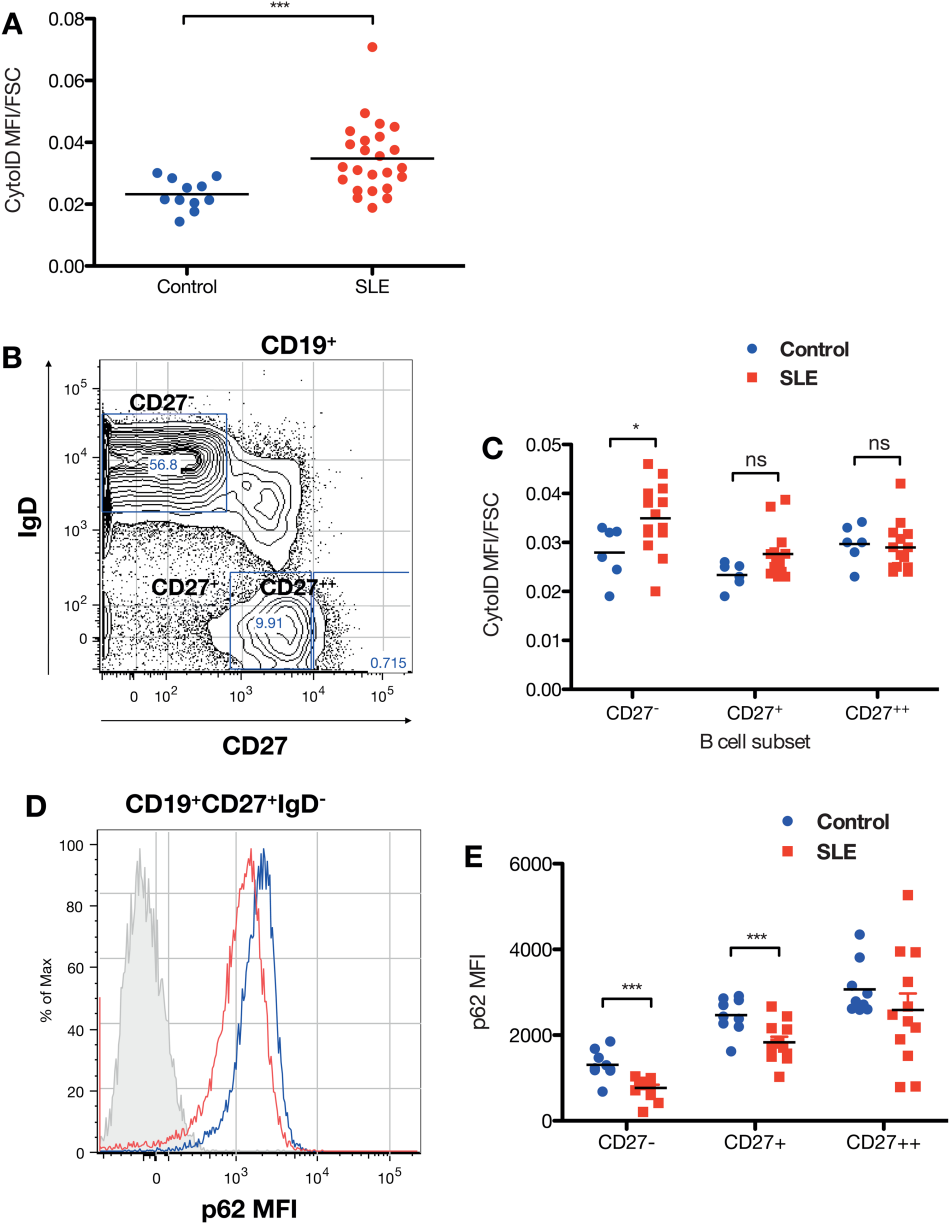
Differential autophagy in human B cell subsets. (A) The increase in autophagosome density seen in CD19^+^ B cells with multispectral imaging flow cytometry is also demonstrated using autophagosomotropic dye. (B) Gating strategy for naïve (CD27^−^IgD^+^), memory (CD27^+^IgD^−^), and plasmablast (CD27^++^IgD^−^) B cells, and differential autophagy by subset (C). (D) Example histogram of p62 MFI in naïve B cells of systemic lupus erythematosus (red line) compared with control (blue line). (E) Differential p62 expression in B cell subsets. *p<0.05; ***p<0.001.

Analysis of total CD19^+^ B cells using CytoID confirmed the increase in autophagosome load in SLE (figure 4A). When analysed by B cell subset (CD19^+^CD27^−^ naïve, CD19^+^CD27^+^ memory and CD19^+/−^CD27^++^ plasmablast) we found that in both healthy controls and patients, dye uptake was greatest in naïve B cells, with lower levels in memory B and plasmablasts (figure 4C). Autophagosome density was significantly higher in naïve B cells, with lesser increases in memory B and plasmablasts. p62 levels, which are inversely correlated with autophagy, mirrored these results, demonstrating higher turnover of this autophagy substrate in naïve B cells in both controls and cases, but by this assay autophagy was increased in SLE in all B cell subsets, although to a much lesser extent in plasmablasts (figure 4E).

Results from the NZB/W and Vav-atg7^−/−^ mice suggest that autophagy may play a role in transitioning through tolerance checkpoints, and may therefore allow autoreactive B cells to survive. The human disease data demonstrate that autophagy is differentially activated in the naïve B cell compartment, a developmental stage during which there is a failure to remove autoreactive cells in lupus.^26^ We next sought to determine if autophagy was required at a later stage in B cell development, the survival and formation of antibody-secreting cells.

### Autophagy is required for efficient plasma cell development

**Figure 5:**
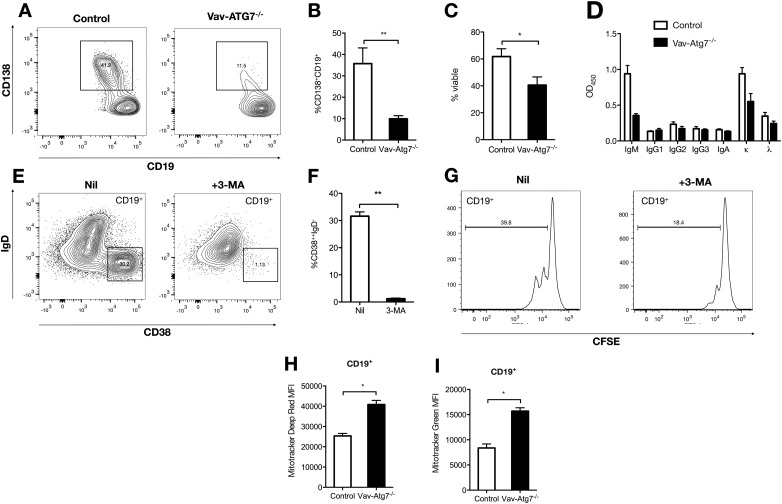
Effects of autophagy inhibition on plasma cell differentiation. (A) B cells from Vav-Atg7^−/−^ or control mice were stimulated with LPS (10 μg/mL) and IL-4 (10 ng/mL) for 72 h. The percentage CD19^+^CD138^+^ plasma cells is shown (B). There was decreased viability following the culture period, as assessed by exclusion of viability dye (C), and decreased secretion of IgM antibody (D). (E) B cells from healthy human donors were stimulated with ODN2006 (5 μM), IL-10 (50 ng/mL), IL-15 (10 ng/mL), and monoclonal mouse anti-CD40 L (1 μg/mL) for 72 h in the presence or absence of the autophagy inhibitor 3-methyladenine (5 mM). The percentage CD19^+^IgD^−^CD38^++^ plasmablasts following culture is shown (F), as is cell proliferation by carboxyfluorescein diacetate succinimidyl ester dilution (G). There is higher mitochondrial mass in Vav-Atg7^−/−^ B cells, measured by MitoTracker Deep Red and Green (H) and (I), compared with control mice. (A–D) are representative of two independent experiments with n=5 mice per group. (E and G) are representative of three individual healthy donors. (H-I) n=5 per group. *p<0.05; **p<0.01.

To determine the requirement of autophagy for terminal B cell differentiation into plasma cells, the ability of Atg7 deficient B cells to form plasma cells in vitro was assessed. Isolated splenic B cells were induced to differentiate using lipopolysaccharide (LPS) and IL-4. There was reduced cell survival during the culture period in the Atg7^−/−^ B cells, and of the surviving cells there were substantially fewer CD138^+^ plasma cells (figure 5A–D), with less immunoglobulin production. Mitochondrial density in freshly isolated Atg7^−/−^ B cells was significantly higher than controls (figure 5H,I).

We next examined the effect of in vitro inhibition of autophagy on human B cell differentiation (figure 5E–G). Isolated CD19^+^ B cells were stimulated with the type A CpG ODN2006, anti-CD40, IL-10 and IL-15, a regime that induces CD38^hi^ plasmablast development after 4 days of culture.^27^ Autophagy was modulated by 3-methyladenine (3-MA), a class III selective phosphoinositide-3-kinase (PI3K) inhibitor that blocks the kinase Vps34, which is essential for the initiation of autophagosome development.^28^ There was a dramatic reduction in plasmablast formation with 3-MA treatment, with associated reduction in cell proliferation measured by CFSE dilution. Recent work has indicated that in some circumstances 3-MA may in fact induce autophagy.^29^ However, we confirmed an inhibitory effect in our experimental system (see online supplementary figure S2).

### Autophagy is downregulated by B cell survival signals and SLE serum

**Figure 6:**
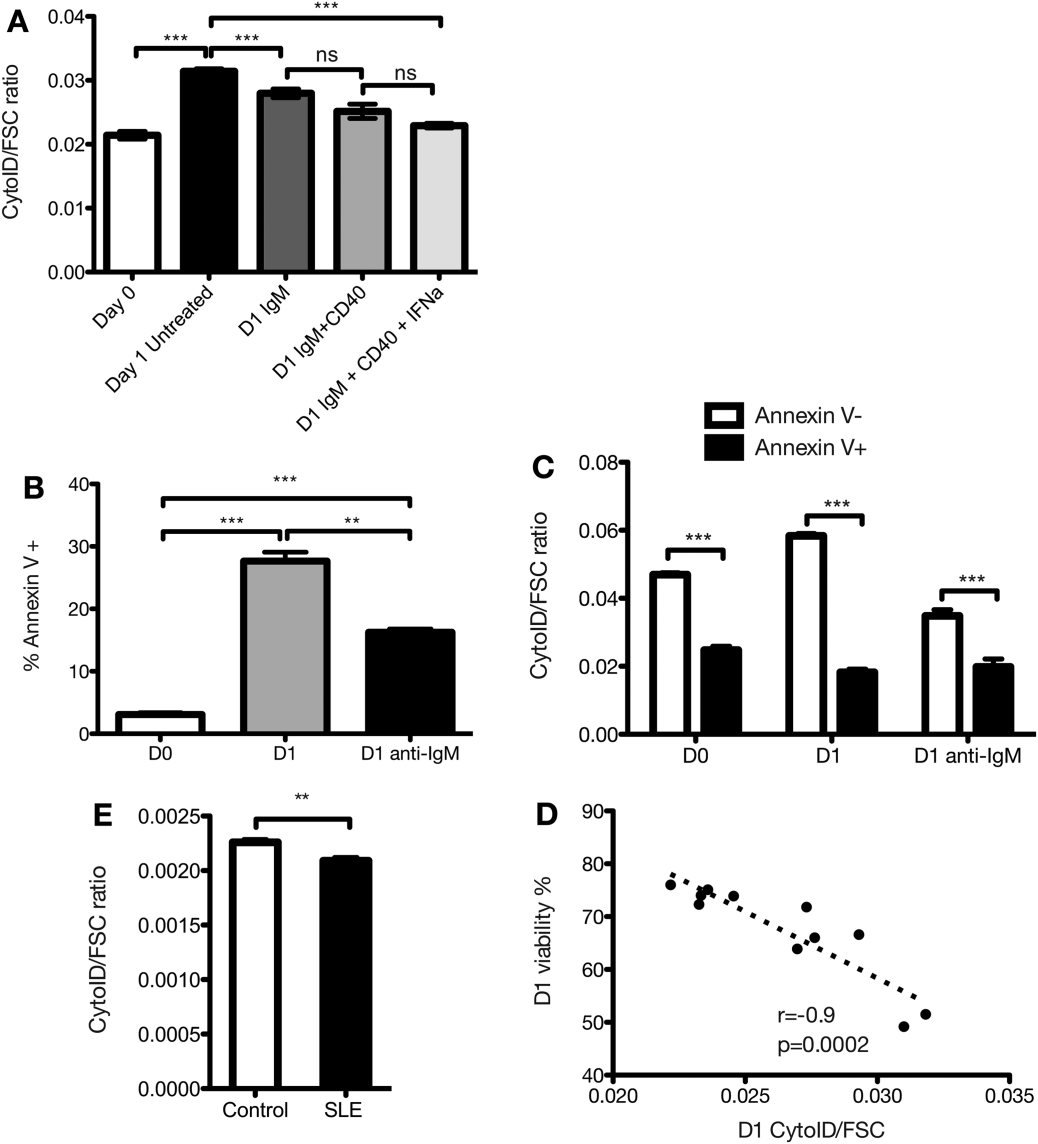
Effect of in vitro stimulation of human B cells on autophagy. (A) Human B cells were isolated and stimulated with combinations of anti-IgM, anti-CD40 and interferon-α, then stained with CytoID. (B) Annexin V staining in B cells, cultured with and without anti-IgM, and CytoID uptake in cells grouped as annexin V positive or negative (C). (D) Correlation between autophagy activation and cell viability, as assessed by viability dye exclusion. (E) Effect of B cell culture in healthy control or systemic lupus erythematosus patient serum on autophagy. Isolated B cells were cultured in RPMI supplemented with 10% human serum for 4 h, then stained with CytoID. Data illustrate the effects of serum from three patients and three healthy controls. Panels A–E are representative of three or more independent experiments. *p<0.05; **p<0.01; ***p<0.001.

We next determined the effect of in vitro stimulation of human B cells with sIgM ligation, with or without CD40 ligation or interferon-α stimulation. We found that autophagy was maximally activated in untreated primary B cells following 24 h culture, with additive reductions on the addition of sIgM binding, CD40 ligation and interferon-α treatment (figure 6A). Interestingly, while apoptosis (measured by annexin V binding) was elevated alongside autophagy (figure 6B,C), there was a degree of mutual exclusivity, in that annexin V^+^ cells had markedly lower levels of autophagy than non-apoptotic cells. This is in keeping with the sequestration of Beclin-1 by interaction with Bcl-2,^30^ and suggestive of activation of apoptosis following the failure of autophagy. The degree of activation of autophagy was inversely correlated with cell survival 24 h later (figure 6D). We also found statistically significant but relatively minor suppression of autophagy in primary B cells by incubation with serum from patients with SLE, compared with serum from healthy donor controls (figure 6E).

## Discussion

We have presented data implicating autophagy in the pathogenesis of human and murine SLE, and demonstrated a requirement for autophagy in B cell survival and differentiation, particularly during early development, and in the formation of plasmablasts. Whilst autophagy has been explored in T lymphocytes, its role in the homeostasis of B cells has received less attention. SLE is a disease characterised by breach of multiple tolerance mechanisms, with the result that autoreactive B cells survive and proliferate to form plasma cells producing high-affinity autoantibodies.^11^

During normal B cell development, *Igh* locus recombination frequently (up to 75% of immature B cells in humans)^31^ results in the production of autoreactive BCRs, which are tested for binding to self-antigens at the transition from pre-B to immature B cell stage, through pre-BCR signalling. This represents an initial tolerance checkpoint, with the vast majority of the newly generated B cell repertoire not surviving to exit the BM,^32^ with cell death occurring through apoptosis. Indeed, levels of the anti-apoptotic molecule Bcl-2 increase as developing B cells transition from immature to mature stages.^33^ Bcl-2 negatively regulates autophagy through its interaction with Beclin-1,^30^ and B cell developmental stages with low Bcl-2 expression coincide with higher levels of autophagy.

However, the resting, mature peripheral B cell pool in autophagy deficiency is largely normal. Autophagy may therefore be activated during early B cell development as a means to survive pro-apoptotic stimuli associated with the generation of a self-reactive or otherwise dysfunctional BCR.

We found that in NZB/W mice, autophagy was maximally increased compared with B6 control mice during early B cell development, at the pre-B to mature B cell stage.

Analysis of autophagy in peripheral B cells of patients with SLE demonstrated maximal activation in naïve B cells, which encounter a tolerance checkpoint following egress from the BM, which has been shown to be defective in SLE.^26^ We therefore propose that enhanced autophagy at this stage may allow B cells with autoreactive BCRs to escape physiological deletion. Stimulation of human B cells in vitro demonstrates that autophagy is activated in the absence of survival signals, but is reduced additively with BCR stimulation, CD40 ligation and interferon-α. We found a degree of mutual exclusivity between autophagy and apoptosis, suggesting activation of programmed cell death if autophagy failed. These results support previous observations that autophagy is induced in B cells in the absence of co-stimulation, a situation that leads to cell death.^12^

Interestingly, we found an age independent increase in autophagy in the B cells of the lupus prone NZB/W F_1_ strain, with significantly more autophagy than the control B6 strain even at a young age, 4 weeks, before the onset of disease.^19^ NZB/W mice have a genetically determined defect in B cell activation, with excessive polyclonal IgM production from shortly after birth, and impaired tolerance induction.^20^ ^21^ The function of autophagy activation in these mice, as with the human SLE data, may represent an attempt by autoreactive B cells to survive deletion. However, to what extent autophagy is required for disease development is an outstanding question.

We also demonstrated an important role for autophagy in plasmablast differentiation, in both Atg7^−/−^ and human B cells. Our results confirm similar observations in Atg5^−/−^ models,^6^ ^7^ with the advantage of knockout of a gene without known functions outside of autophagy. We found moderately decreased viability in Atg7^−/−^ B cells following stimulation, but a marked failure of differentiation into plasma cells, associated with reduced secretion of IgM. The transition from resting B cell to plasma cell, capable of secretion of large quantities of immunoglobulin, generates intense metabolic stress, and is dependent on the induction of the unfolded protein response, triggered from the ER.^34^ ^35^ The ER is expanded in autophagy deficiency,^7^ ^36^ and the enhanced ER stress associated with this may be inhibitory to plasma cell differentiation.^37^ Similarly, ineffective clearance of defective mitochondria by impaired mitophagy predisposes cells to apoptosis,^38^ and this may represent another explanation for our findings. In SLE, the plasmablast population is often markedly expanded, and is correlated with disease activity.^39^ Pharmacological inhibition of autophagy restricts human plasmablast differentiation in vitro, as was seen with murine Atg7^−/−^ cells.

Autophagy therefore presents a potential therapeutic target in SLE, and may be a clinical relevant mechanism of action of the commonly used immunomodulatory anti-malarial hydroxychloroquine, which is an inhibitor of autophagy by raising lysosomal pH and therefore preventing autophagosome–lysosome fusion.^40^ Similarly, many pharmaceuticals approved in the European Union and USA, and in regular clinical use for alternative indications, inhibit autophagy and may therefore be novel treatments for SLE.^41^

## Supplementary Material

Web supplement

Web table S1
