# Supplementary material for: Autophagy is activated in systemic lupus erythematosus and required for plasmablast development
Source: Ann Rheum Dis. 2014 Jan 13;74(5):912–20. doi: 10.1136/annrheumdis-2013-204343 (PMC4152192; doi:10.1136/annrheumdis-2013-204343)
Supplement: Web supplement [file annrheumdis-2013-204343-s1.pdf]

Supplementary Methods

Multispectral imaging flow cytometric lysosome-autophagosome co-localization

The protocol used for dual staining of lysosomes and autophagosomes was adapted from Phadwal *et al*.(1) 5×10^6 PBMC were used per sample. The cells were first stained with 0.5µl LysoID Red (Enzo, UK) in 500µl RPMI 1640 (containing 10% FCS, 2mM L-glutamine, but no antibiotics) at 37°C for 30 minutes. They were then washed once in phosphate-buffered saline containing 0.5% bovine serum albumin, and then stained for surface antigens, fixed, and stained for intracellular antigens as described in the main Methods section. To quantify co-localization, the Bright Detail Similarity (BDS) algorithm in Amnis IDEAS 6 was used to measure the degree of similarity between background-subtracted bright details ≤3 pixels in diameter from the LysoID and LC3-FITC channels. BDS is the log-transformed Pearson's correlation co-efficient between the two analysed images, following appropriate masking.

Immunoblotting

Cells were lysed in ice-cold RIPA buffer (Sigma, UK), supplemented with protease and phosphatase inhibitors (Complete Mini and PhoSTOP, Roche, UK) for 15 minutes, then protein concentration was determined using the bicinchoninic acid method (BCA Assay Kit, Thermo Scientific).

Twenty-five µg protein per well were separated by SDS-PAGE and transferred onto a PVDF (Immobilon P, Merck Millipore, UK) membrane, then probed overnight with

polyclonal rabbit anti-LC3 1:4000 (Novus, US), or rabbit monoclonal anti-β-actin 1:20000 (Cell Signaling Technology). Blots were developed with Luminata Forte ECL (Merck Millipore, UK). Band intensities were quantified using ImageJ (NIH).

Supplementary Figure Legends

Figure S1. Multispectral imaging flow cytometric lysosome-autophagosome co-localization. Co-localization between autophagosomes and lysosomes in CD19^{+} B cells from SLE patients and healthy controls. Representative images of cells with low and high levels of co-localization (BDS) **(A)**. There was intact autophagosome-lysosome co-localization and therefore flux in SLE patients compared with healthy controls, with a trend towards increased co-localization in disease **(B)**.

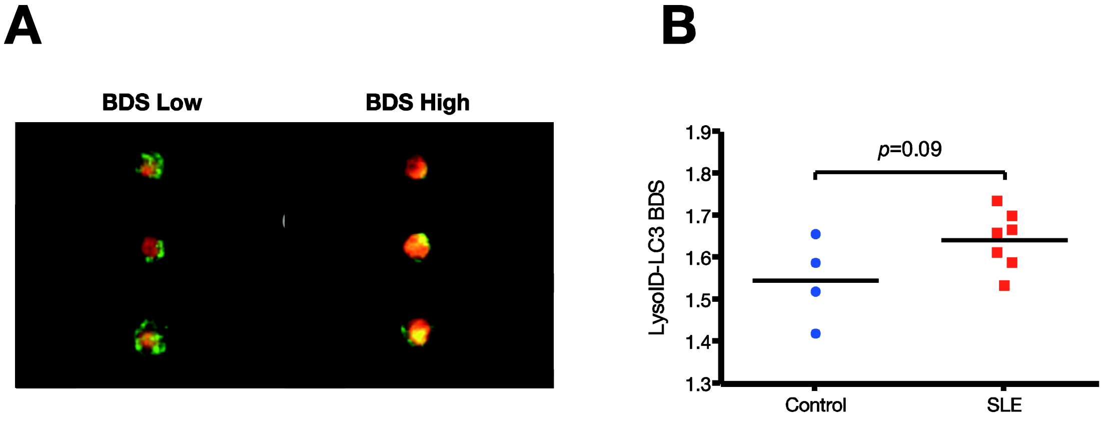

Figure S1

Figure S2. 3-MA inhibits autophagy during B cell stimulation and culture. B cells from healthy human donors were stimulated with ODN2006 (5μM), IL-10 (50ng/ml), IL-15 (10ng/ml), and monoclonal mouse anti-CD40L (1μg/ml) for 72 hours in the presence or absence of the autophagy inhibitor 3-methyladenine (5mM). During the last hour of culture, bafilomycin A_1 to a concentration of 20nM was added. A representative western blot is shown **(A)**, with LC3-II:β-actin ratios from 2 independent experiments. Error bars indicate standard error of the mean **(B)**.

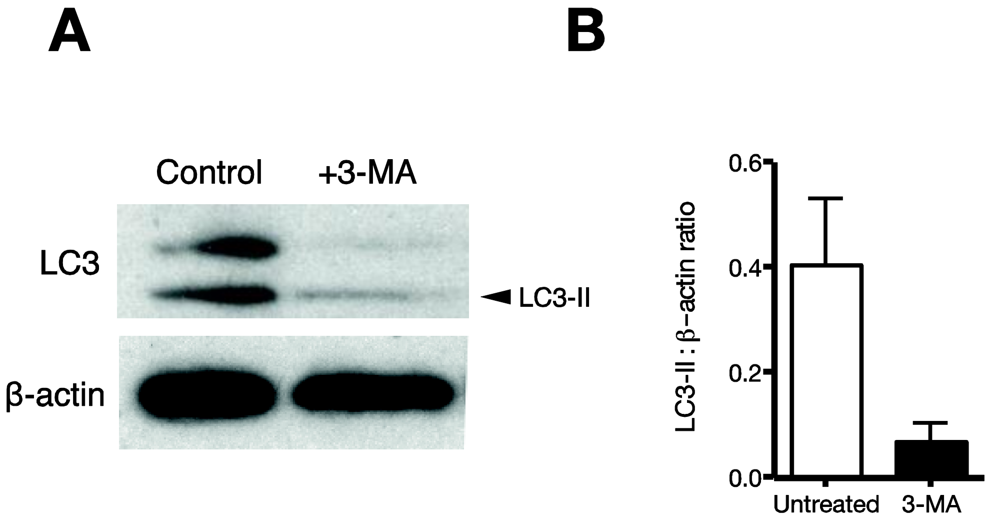

Figure S2

1. Phadwal K, Alegre-Abarrategui J, Watson AS, Pike L, Anbalagan S, Hammond EM, et al. A novel method for autophagy detection in primary cells: Impaired levels of macroautophagy in immunosenescent T cells. Autophagy. 2012 Apr 1;8(4).
