# Supplementary material for: Autophagy is activated in systemic lupus erythematosus and required for plasmablast development
Source: Ann Rheum Dis. 2014 Jan 13;74(5):912–20. doi: 10.1136/annrheumdis-2013-204343 (PMC4152192; doi:10.1136/annrheumdis-2013-204343)
Supplement: Web table S1 [file annrheumdis-2013-204343-s2.pdf]

Table S1. Patient Characteristics

**Patient number**	**Sex**	**Age**	**Diagnostic Clinical Features (ACR)**	**Antibodies**	**C3/4**	**SLEDAI**	**Medication**
1	F	33	Arthritis, Immunologic, Pleurisy, ANA	Anti-dsDNA	Low	10	Aza, Pred
2	F	63	Malar rash, Oral ulcers, ANA Discoid		Normal	6	HCQ
3	F	23	Photosensitivity, ANA, Immunologic, Lymphopenia	Anti-dsDNA	Normal	4	MMF, HCQ
4	F	25	Renal, ANA, Immunologic, Haematologic	Anti-dsDNA	Normal	6	HCQ, Pred
5	F	55	Arthritis, ANA, Renal, Haematologic	Anti-Ro	Normal	4	Pred
6	F	45	Renal, ANA, Immunologic, Haematologic	Anti-Sm, anti-RNP	Normal	8	MMF, Pred
7	F	26	Arthritis, Mouth ulcers, ANA, Immunologic	Anti-dsDNA	Normal	10	HCQ
8	F	54	Haematologic, ANA, Immunologic, Arthrtis	Anti-dsDNA	Normal	5	HCQ, Pred
9	F	28	Arthritis, Mouth ulcers, ANA, Immunologic	Anti-cardiolipin	Normal	6	HCQ, Pred

10	F	43	Arthritis, Mouth ulcers, ANA, Arthritis		Normal	6	HCQ
11	F	34	Renal, Photosensitivity, ANA, Malar rash		Normal	0	MMF, HCQ
12	F	31	Arthritis, Mouth ulcers, Immunologic, ANA	Anti-dsDNA, anti-Ro	Low	12	MMF, Pred
13	F	42	Malar rash, Haematologic, Arthritis, Immunologic, Photosensitivity	Anti-Ro, anti-RNP	Normal	3	MMF, HCQ
14	F	39	Haematologic, Arthritis, ANA, Immunologic	Anti-RNP, anti-cardiolipin	Normal	1	MTX, HCQ
15	F	28	Immunologic, ANA, Renal, Cerebral	Anti-dsDNA	Normal	4	MMF, HCQ
16	F	46	Photosensitivity, Mouth ulcers, Immunologic, ANA	ANA, Ro	Low	10	MMF, Pred
17	F	38	Photosensitivity, Renal, Arthritis, ANA, Immunologic	Anti-dsDNA, anti-Ro, anti-RNP	Low	8	Pred, HCQ
18	F	47	Renal, ANA, Immunologic, Haematologic	Anti-Ro, anti-dsDNA	Low	8	Aza, Pred, HCQ
19	F	69	Malar rash, ANA, Immunologic, Haematologic	Anti-Ro/La, anti-dsDNA	Low	8	Pred, HCQ
20	F	62	ANA, Arthritis, Photosensitivity,		Normal	7	Aza, Pred, HCQ

			Renal				
21	F	22	Arthritis, Malar rash, Pleuritis, ANA, Immunologic, Haematologic	Anti-dsDNA	Low	13	MMF, HCQ
22	F	36	Immunologic, ANA, Malar rash, Mouth ulcers	Anti-dsDNA, Ro, RNP	Normal	6	MMF, MTX, Pred
23	F	27	Renal, ANA, Immunologic, Arthritis, Photosensitivity	Anti-RNP, anti-Ro, anti-Sm, anti-dsDNA	Low	12	MMF, Pred
24	F	41	Renal, ANA, Immunologic, Mouth ulcers	Anti-dsDNA	Low	12	Aza, Pred, HCQ
25	M	32	Arthritis, Discoid rash, Malar rash, ANA	Anti-Ro	Low	11	Pred
26	F	27	Arthritis, Photosensitivity, ANA, Immunologic	Anti-dsDNA, anti-Ro	Normal	8	Pred
27	F	29	ANA, Mouth ulcers, Malar rash, Renal, Haematologic, Neurologic	Anti-dsDNA, anti-Ro, anti-Sm	Low	14	Pred, MMF
28	F	54	ANA, Arthritis, Photosensitivity, Renal, Immunologic	Anti-RNP, anti-Sm	Low	8	HCQ
29	F	44	ANA, Cerebral, Arthritis, Photosensitivity		N	0	Aza, Pred, HCQ

30	M	23	ANA, Malar rash, Immunologic, Renal	Anti-dsDNA, anti-Ro	Low	8	Pred, MMF
31	F	51	ANA, Immunologic, Renal, Pericarditis, Malar rash	Anti-dsDNA, anti-Ro/La	Low	6	MMF, Pred, HCQ
32	M	23	ANA, Malar rash, Renal, Photosensitivity, Immunologic	Anti-dsDNA	Low	12	MMF, Pred, HCQ
33	F	33	Malar rash, Photosensitivity, Arthritis, ANA, Immunologic	Anti-dsDNA, anti-Ro	Low	10	HCQ
34	F	41	ANA, Arthritis, Immunologic, Photosensitivity	Anti-dsDNA, anti-Ro, anti-Sm	Low	8	MMF, HCQ
35	F	43	Renal, Mouth ulcers, Immunologic, Malar rash	Anti-Ro, anti-RNP	Normal	8	MMF, Pred, HCQ
36	M		Malar rash, Arthritis, ANA, Immunologic	Anti-dsDNA	Normal	6	Nil
37	F	29	ANA, Haematologic, Immunologic, Arthritis	Anti-dsDNA, anti-Ro, anti-RNP	Low	6	Pred, Mepacrine
38	F	30	ANA, Arthritis, Photosensitivity, Malar rash		Normal	0	HCQ

39	F	30	Arthritis, Malar rash, Mouth ulcers, Immunologic	Anti-dsDNA, anti-Ro	Low	12	HCQ
40	F	36	ANA, Mouth ulcers, Photosensitivity, Arthritis		Normal	4	HCQ
41	F	24	ANA, Mouth ulcers, Malar rash, Immunologic	Anti-dsDNA, anti-Ro	Low	8	HCQ, Pred
42	M	52	ANA, Photosensivity, Arthritis, Mouth ulcers		Normal	0	HCQ
43	F	29	ANA, Discoid, Mouth ulcers, Arthritis, Immunologic	anti-dsDNA	Low	9	HCQ

Abbreviations: Aza – azathioprine; Pred – prednisolone; HCQ - hydroxychloroquine, MMF - mycophenolate mofetil
